# Interim clinical trial analysis of intraoperative mass spectrometry for breast cancer surgery

**DOI:** 10.1038/s41523-021-00318-5

**Published:** 2021-09-09

**Authors:** Sankha S. Basu, Sylwia A. Stopka, Walid M. Abdelmoula, Elizabeth C. Randall, Begoña Gimenez-Cassina Lopez, Michael S. Regan, David Calligaris, Fake F. Lu, Isaiah Norton, Melissa A. Mallory, Sandro Santagata, Deborah A. Dillon, Mehra Golshan, Nathalie Y. R. Agar

**Affiliations:** 1 Department of Pathology, Brigham and Women’s Hospital, Harvard Medical School, Boston, MA USA; 2 Department of Neurosurgery, Brigham and Women’s Hospital, Harvard Medical School, Boston, MA USA; 3 Department of Radiology, Brigham and Women’s Hospital, Harvard Medical School, Boston, MA USA; 4 Department of Surgery, Brigham and Women’s Hospital, Harvard Medical School, Boston, MA USA; 5 Department of Cancer Biology, Dana-Farber Cancer Institute, Harvard Medical School, Boston, MA USA; 6 Present Address: Yale Cancer Center, Department of Surgery, New Haven, CT USA

**Keywords:** Tumour biomarkers, Breast cancer

## Abstract

Optimal resection of breast tumors requires removing cancer with a rim of normal tissue while preserving uninvolved regions of the breast. Surgical and pathological techniques that permit rapid molecular characterization of tissue could facilitate such resections. Mass spectrometry (MS) is increasingly used in the research setting to detect and classify tumors and has the potential to detect cancer at surgical margins. Here, we describe the ex vivo intraoperative clinical application of MS using a liquid micro-junction surface sample probe (LMJ-SSP) to assess breast cancer margins. In a midpoint analysis of a registered clinical trial, surgical specimens from 21 women with treatment naïve invasive breast cancer were prospectively collected and analyzed at the time of surgery with subsequent histopathological determination. Normal and tumor breast specimens from the lumpectomy resected by the surgeon were smeared onto glass slides for rapid analysis. Lipidomic profiles were acquired from these specimens using LMJ-SSP MS in negative ionization mode within the operating suite and post-surgery analysis of the data revealed five candidate ions separating tumor from healthy tissue in this limited dataset. More data is required before considering the ions as candidate markers. Here, we present an application of ambient MS within the operating room to analyze breast cancer tissue and surgical margins. Lessons learned from these initial promising studies are being used to further evaluate the five candidate biomarkers and to further refine and optimize intraoperative MS as a tool for surgical guidance in breast cancer.

## Introduction

In 2019, nearly 2.4 million women were diagnosed with breast cancer worldwide; 250,000 of these women will be diagnosed in the United States^1^. Despite advances in drug therapy and radiation, surgery remains the initial treatment modality of choice for the vast majority of early-stage breast cancers^2^. It is now well established that breast-conserving surgery (BCS) with whole breast radiation provides the same long-term survival as mastectomy^3,4^. Effective BCS involves the removal of breast cancer with clear margins. Positive margins contribute to increased local recurrence rates, and subsequent re-excision can delay adjuvant therapies, diminish aesthetic outcomes, increase infection rates, and increase health care costs and patient stress^5^. Several studies have demonstrated re-excision rates as high as 20–40%^6,7^. Although there have been some improvements to help guide surgeons prior to or during surgery^8–10^, the tools that are available to the surgeon remain limited. Even intraoperative MRI, which provides considerable benefit in neurosurgery^11,12^, has proven challenging to effectively implement in BCS due to positional shifts and tissue distortion that occur during resection^13,14^. Currently, there is no reliable intraoperative method of determining if a margin is clear in real-time or during the operative setting. Surgeons rely on the final histopathology report that typically takes 4–10 days for final assessment. This leads to the significant minority of women who need re-excision to achieve a clear margin. Therefore, methods that allow adequate sensitivity and specificity for tumor detection, while decreasing turnaround time are greatly desired.

Mass spectrometry (MS) is a maturing analytical technique that can be used to measure a wide range of endogenous molecules in a variety of different biological matrices without the need for molecular labeling. Although there are numerous types of MS platforms, they all differentiate molecules based on their mass-to-charge ratios (*m/z*). The past two decades have brought a substantial increase in the utilization of MS-based platforms in the clinical space, including gas chromatography-MS and tandem-MS for newborn metabolic screens^15,16^, liquid chromatography-tandem MS (LC-MS/MS) for therapeutic drug monitoring (TDM)^17^, matrix-assisted laser desorption-time of flight (MALDI-TOF) for microbial identification^18^, and more recently laser microdissection proteomics for characterization of amyloids^19,20^. Although each of these platforms allows for the reproducible measurement and identification of specific analytes, each of the methods involves extensive sample preparation and lengthy analysis time, thereby limiting their use for surgical pathology analysis, especially in the operating room where rapid feedback is desired.

Alternatively, ambient ionization MS allows rapid biochemical characterization under atmospheric conditions, through the direct analysis of tissue, thus requiring minimal sample preparation^21^. The short turnaround time of these methods makes them particularly attractive for intraoperative settings, where real-time feedback is needed to optimize surgical management^22–25^. Several key factors are needed to be addressed when introducing a mass spectrometer into a surgical suite including institutional compliances and institutional review board (IRB) protocols, and eventual FDA approval for broader clinical implementation. Over the years, a few research clinical surgery settings have successfully implemented MS-based IRB protocols in the surgical suite^26^, for both ex vivo and in vivo intraoperative tumor analysis. Moreover, the MassSpec Pen was recently coupled to the da Vinci Surgical System for porcine liver and stomach tissue classification^27^. However, a significant amount of work is still needed to seemly incorporate MS methods for in vivo human analysis and even establish a more facile communication between the surgical and scientist teams. Both technological advancements and clinical space accessibility are critically important to achieve this common goal. There are a wide variety of ambient MS ionization methods currently available. One such method is desorption electrospray ionization (DESI) MS, which we have used to both detect 2-hydroxyglurate (2-HG)^26^, an oncometabolite found in high concentrations in isocitrate dehydrogenase (IDH) mutant gliomas, and classify various brain tumors based on their lipid composition^28^. In addition, we have shown that DESI MS can be used to characterize metabolomic profiles of breast tissues for the detection and identification of breast carcinoma in frozen tissue sections of mastectomy specimens^29^. While DESI MS analysis of fresh brain tissue and frozen breast tissue specimens provides a suitable signal, using this method to analyze fresh breast tissues has been limited due to the high tissue adiposity and physical properties of the breast tissue itself. As such, we have investigated additional sampling platforms to improve fresh breast tissue analysis.

Among ambient ionization techniques^21^, liquid microjunction surface sampling probe (LMJ-SSP) coupled with electrospray ionization^30^ provides an opportunity to improve both processing speed and analyte extraction, features that are desired for intraoperative breast tissue analysis. LMJ-SSP MS involves uniform and continuous microfluidic solvent dispensing, followed by aspiration of the solvent and solvent-extracted analytes and injection into the mass spectrometer using electrospray ionization^31^. Unlike direct ambient methods such as DESI, the LMJ-SSP allows for the longer application of extraction solvent on the tissue, thereby improving the extraction of specific analytes. Here, we describe a clinical application of intraoperative LMJ-SSP MS. This analysis was performed on fresh surgical breast samples collected and analyzed within the Advanced Multimodality Image Guided Operating (AMIGO) suite in the context of a registered clinical trial including the evaluation of MS for breast cancer surgery (ClinicalTrials.gov Identifier: NCT02335671.

## Results

### Invasive carcinoma surgical cases

In this study, we used intraoperative MS to analyze specimens from twenty-one patients with early-stage invasive breast carcinoma. As summarized in Table 1, the tumor features encountered in clinical practice were considerably diverse and included ductal (*n* = 11), lobular (*n* = 2), mixed ductal and lobular (*n* = 6), tubular (*n* = 1), and mucinous micropapillary (*n* = 1) subtypes of breast cancer, with the histologic grades ranging from well-differentiated to poorly-differentiated. In 15 of 21 cases, either ductal carcinoma in situ (DCIS) or lobular carcinoma in situ (LCIS) was also present within the specimen. Although we were able to collect and analyze six surgical margins (superior, inferior, anterior, posterior, medial, and lateral) from each of the excised lumpectomy specimens, we were only able to analyze a tumor sample for 10 of the 21 cases. These cases included specimens in which there was enough tumor available for both clinical care and research trial allocation (tumors greater than 1.5 cm in diameter according to protocol criteria). For two of the cases (cases 13 and 14), fresh surgical specimens were also analyzed using Stimulated Raman Scattering (SRS) and Second-Harmonic Generation (SHG) microscopy.

**Table 1:** Diagnostic characteristics from the initial 21 surgical cases with intraoperative MS analysis.

	Invasive carcinoma type	Tumor size (cm)	Overall histological grade	DCIS/LCIS present?	AJCC stage	ER	PR	Her-2
1	Mixed	1.6	Poor	DCIS	T1c N0 (sn)	+	+	−
2	Mixed	1.5	Moderate	LCIS	pT1c N0 (sn)	+	+	−
3	Mixed	1.1 0.25	Moderate	LCIS	pT1c N0 (sn)	+	+	−
4	Ductal	1.4	Well	Mixed	T1c N1a	+	+	+/−
5	Ductal	1	Moderate		pT1b N0 (sn)	+	+	−
6	Ductal	1.6	Well	DCIS	T1c N0 (sn)	+	+	−
7	Ductal	1.7	Poor	DCIS	pT1c N0 (sn)	−	−	−
8	Ductal	2.1	Poor		pT2 N0(sn)	−	−	−
9	Ductal	0.8 0.5	Well	DCIS	pm T1b N0 (sn)	+	+	−
10	Ductal	0.9	Well	DCIS	T1b N0(sn)	+	+	−
11	Ductal	0.6	Moderate	DCIS	pT1b Nx	+	+	−
12	Ductal with spindle features	0.6	Moderate	DCIS	pT1b N1a (sn)	−	−	−
13	Ductal	1.9	Poor	DCIS	pT1c N0 (sn)	+	+	−
14	Ductal	1.9	Moderate		pT1c N0 (sn)	+	+	−
15	Mixed	0.7	Well		pT1b N0	+	+	−
16	Lobular	0.4; <0.1	Moderate	LCIS	pT1b N0 (sn)	+	+	−
17	Tubular	0.8	Well		pT1bN0	+	+	−
18	Lobular	0.9	Well		pT1b N0(sn)	+	+	−
19	Ductal; Mixed	1.3; 0.9	Moderate; Well	DCIS	pT1c N1mi (two loci)	+	+	−
20	Micropapillary mucinous	2.4	Moderate	DCIS	pT2 N0 (sn)	+	+	−
21	Mixed	1.5	Moderate	DCIS	pT1c N0	+	+	−

*DCIS* ductal carcinoma in situ, *LCIS* lobular carcinoma in situ, *LVI* lymphovascular invasion, *ER* estrogen receptor, *PR* progesterone receptor, *Nx* No lymph nodes available for evaluation.

*SRS analyzed.

### Intraoperative MS analysis

**Fig. 1:**
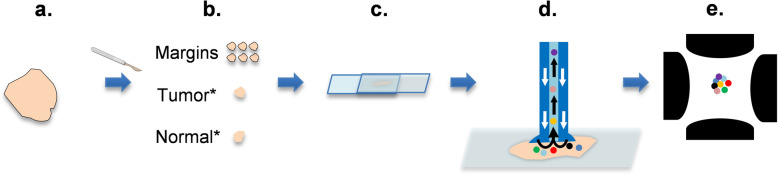
Workflow for intraoperative MS of surgical breast tissue. **a** Lumpectomy, (**b**) tissue allocation, (**c**) tissue smear using squash prep, (**d**) surface extraction using liquid microjunction surface sample probe (LMJ-SSP), and (**e**) metabolite profiles generated using electrospray ionization-ion-trap mass spectrometry. *Tumor and normal samples allocated in the frozen section room when adequate tissue is available for diagnosis.

**Fig. 2:**
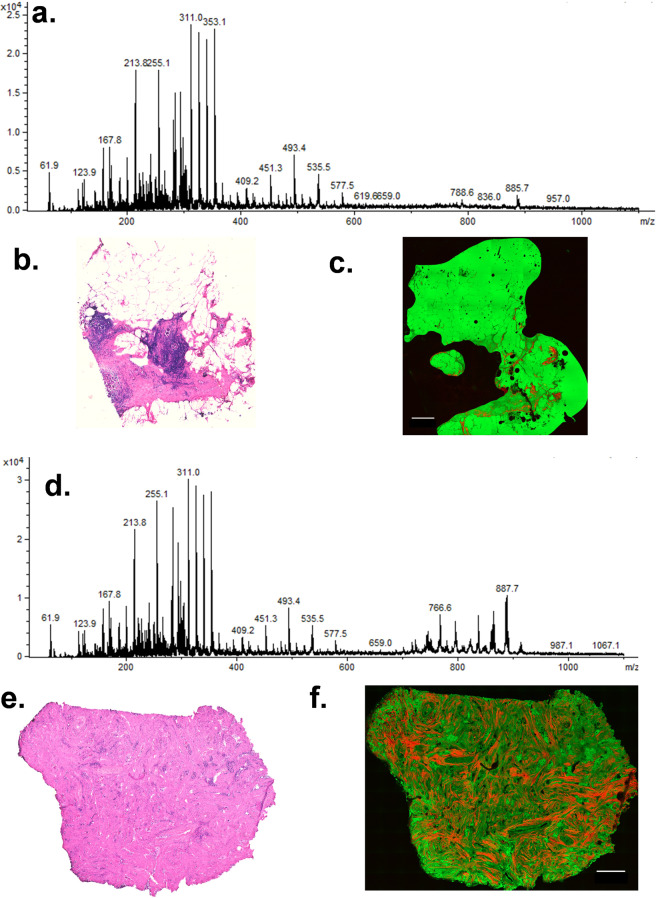
Mass spectra, histological and stimulated Raman scattering microscopy of normal and tumor tissue (case 13). **a** Intraoperative mass spectrum generated from normal (benign) breast tissue. **b** H&E stain of normal tissue. **c** SRS image of sister section. **d** Intraoperative mass spectrum generated from tumor tissue. The scale bar is 250 µm. **e** H&E stain of tumor tissue. **f** SRS image of the parallel section. In SRS image, areas of green correlate with lipids, while orange correlates with collagen. The scale bar is 500 µm.

Specimen freezing and cryo-sectioning prohibit rapid analysis, and, therefore, we used fresh tissue samples in this study and analyzed them using LMJ-SSP MS. The College of American Pathologists (CAP) guidelines were followed, which states a human tissue needs to be processed within 1 h of resection. We adapted our intraoperative MS workflow developed for brain tissue and manually smeared the breast tissue specimen between two glass slides. The overall surgical workflow is presented in Fig. 1. An illustrative example of a mass spectrum collected in the operating room from a fresh surgical specimen, an H&E stain, and an SRS image from a representative normal and tumor sample are presented in Fig. 2. SRS images of the specimens show a significantly higher fat content in the normal sample as compared to the tumor sample, which typically has more collagen, indicative of the more fibrofatty composition of tumors.

**Fig. 3:**
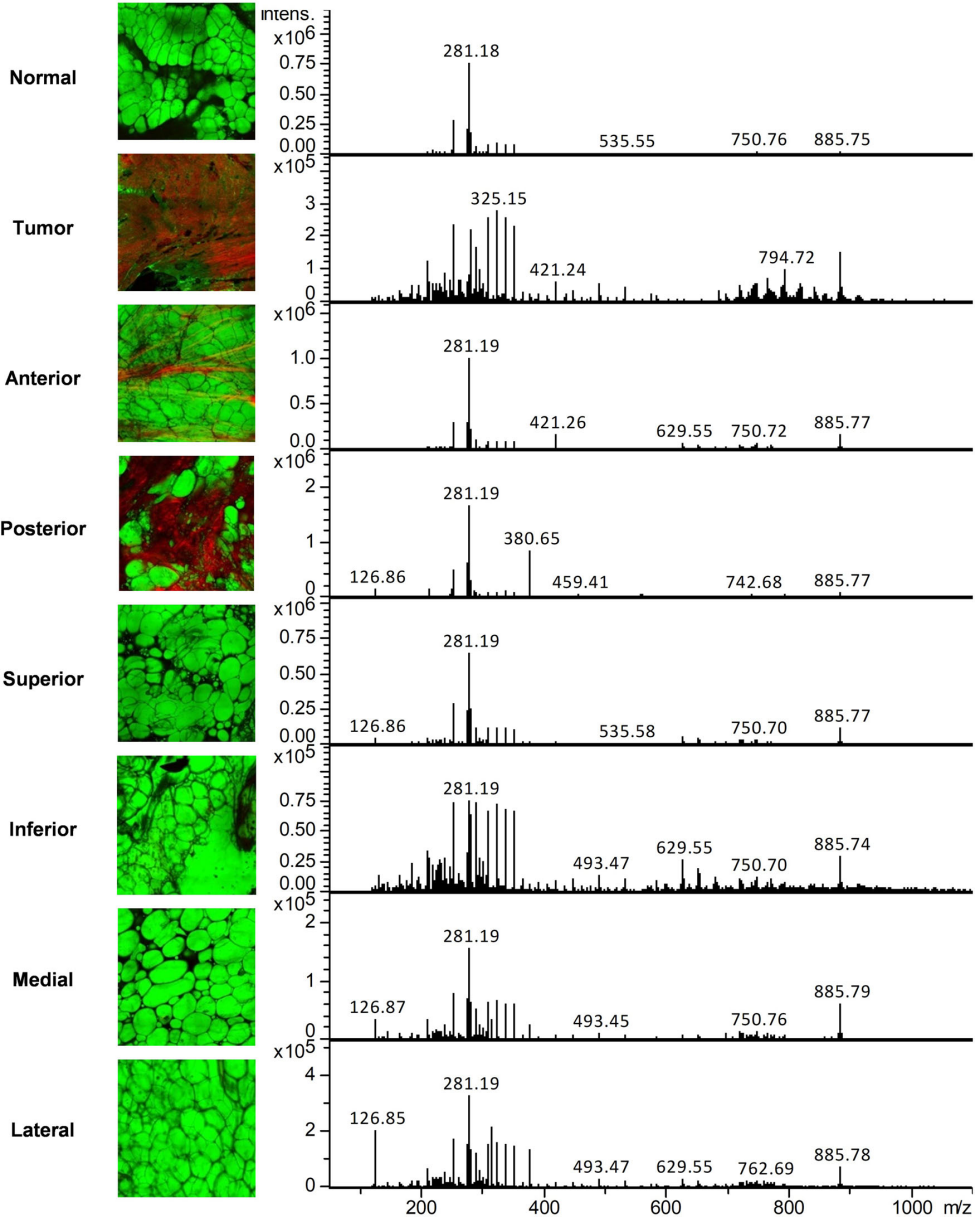
SRS images and intraoperative mass spectra from the tumor, normal, and lumpectomy margins from Case 13. Pseudocolor green: SRS imaging of lipids in adipocytes; pseudocolor red: SHG imaging of collagen. Tumor tissue was featured by significantly reduced lipid contents and increased collagen deposition.

Lipidomic profiles obtained from resections, which included both tumor and normal specimens were compared both qualitatively and quantitatively. As shown in Fig. 2, a representative mass spectrum acquired from a tumor specimen showed a considerably different lipidomic profile than the mass spectrum acquired from a normal adjacent piece of tissue. We noted a much higher density of signals within the lipid range in tumor samples compared to normal breast tissue. Although there were no apparent phospholipids that were higher in all of the tumor specimens, there were certain phospholipids that demonstrated a relative increase in signal intensity in the tumor samples compared to normal. Notably, peaks we had previously observed in frozen tumor tissue using DESI-MS (281.2-oleic acid, 665.6 PA, and 391.4) were not consistently seen in higher concentrations in all tumors when using LMJ-SSP on fresh tissue. However, certain cases such as case 20, showed both a higher peak at 281 and higher phospholipid signals. In addition, we performed an analysis of margin specimens. One representative case highlighting tumor, normal, and margins is presented in Fig. 3. The compilation of all of the margin analyses for all cases will be presented at the completion of the clinical trial evaluation.

### Statistical analysis

**Fig. 4:**
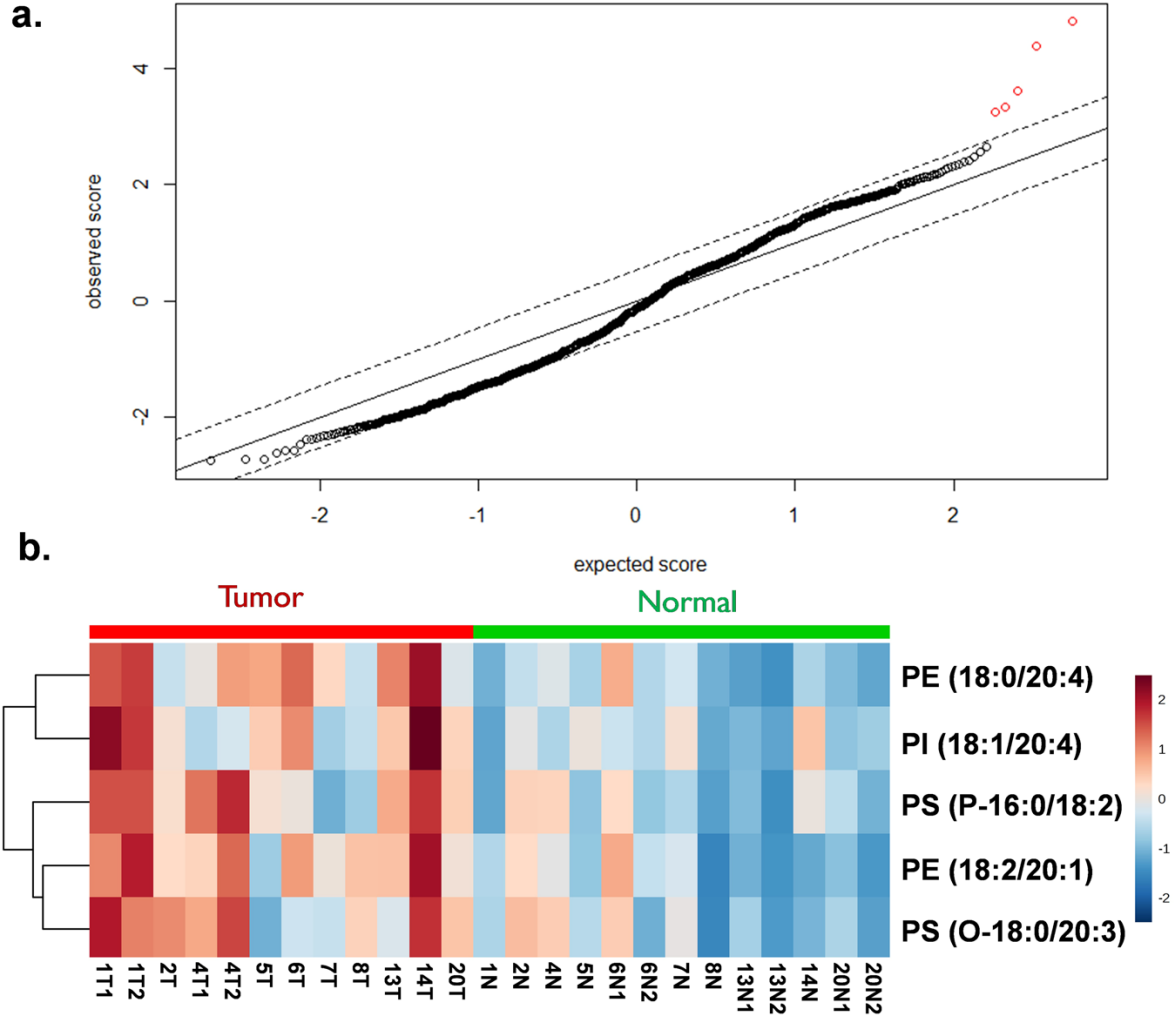
Multivariate analysis. **a** Feature extraction using significance analysis of microarrays (SAM) analysis highlighting five molecules (in red) which represent the most distinguishing features in the tumor compared to normal samples, with a false discovery rate of <0.001 and confidence interval of 90%. **b** The hierarchical map was constructed in an unsupervised and randomized manner, resulting in a heat map demonstrating relative differences in ion intensities of the 5 features between tumor and normal samples.

**Fig. 5:**
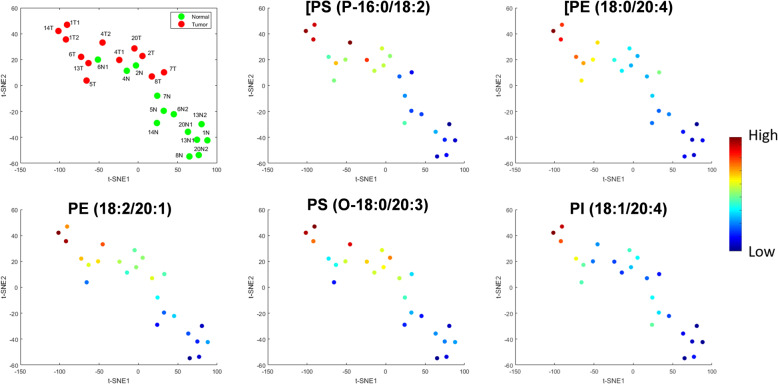
t-distributed stochastic neighbor embedding (t-SNE) demonstrating the placement of each specimen as a data point in an unsupervised manner based on the 5 most discriminant features. Data points are labeled by their case number followed by N (normal) in green or T (tumor) in red, and then by sample number if there was more than one specimen for that designation. The data points were then colored based on the ion intensities of the five significant lipid peaks.

In a preliminary analysis of the limited dataset, we post-surgically performed statistical analyses of the aligned and total ion current (TIC)-normalized spectra (Supplementary Table 1) using the SAM method to find significant discriminant ions. The cutoff threshold of the delta was set to 0.5196 and a 90% confidence interval as it resulted in identifying 5 *m/z* ion features with an FDR < 0.001, designated as the red highlighted points in Fig. 4a. These significant SAM features, based on their ranked significance order are *m/z* = [767.6, 769,6, 743.6, 798.6, 883.7]. These ions are represented as a heat map in Fig. 4b, which demonstrates overall a relatively higher abundance of these molecules in the tumor specimens compared to normal. Dimensionality reduction of these 5 *m/z* SAM features using t-SNE analysis enabled visualization in a two-dimensional space, further revealing an overall separation of tumor and normal (Fig. 5). Although three normal specimens do appear to cluster with the tumor data points in the t-SNE map, histopathological review of adjacent frozen sections of these three normal specimens displayed several regions of hypercellularity consistent with focal involvement by tumor cells in two of the three specimens. Notably, tumors from the two triple-negative breast cancer cases (cases 7 and 8) were found to be closely related (Fig. 5). These data points were further colored based on their ion expression for each of the significant *m/z* SAM features (Fig. 5). Coloring the t-SNE map based on these SAM features reveals structural distribution in which there is a continuous color gradient across the mapped data points.

## Discussion

We describe the application of ex vivo MS analysis of breast cancer tissue in the AMIGO operating suite. On average a breast-conserving therapy is ~82 min at BWH, thus we have developed a workflow in which the research scientist is present during the whole surgery and is immersed in the surgical setting. This is a critical part, to introduce scientists into the surgical suite to understand the communication with the surgical team but also to start becoming an integrated part of analysis during surgery. This role will become extremely important in future studies in which the surgeon could have a probe for in vivo analyses. Thus, all of the ex vivo samplings were performed in the AMIGO suite. The workflow involved (1) enrolling patients and procuring specimens from subjects meeting pre-specified criteria, (2) collecting and processing fresh breast tissue samples in the AMIGO suite, (3) performing intraoperative LMJ-SSP on the smeared samples for mass spectrometry data acquisition, and (4) analyzing mass spectra from surgical specimens after surgery. Each of these aspects presented specific challenges that will be discussed here.

For this initial study, we chose subjects who had newly diagnosed breast cancer who had not received neoadjuvant therapy, thus avoiding the confounding effects of molecular changes induced by treatment with endocrine, targeted, and/or chemotherapeutic agents. Identifying treatment-naive subjects, however, with tumors of adequate size for both clinical care and research analysis (>1.5 cm in diameter) made patient enrollment challenges. In addition to enrollment, sample procurement also presented substantial logistical challenges. To provide optimal clinical care, our approved protocol allowed us to collect six margin specimens from each sample, but tumor tissue only when the tumor measured at least 1.5 cm in the greatest dimension. Consequently, only about half of the specimens in our study had sufficient tumor present to permit the analysis of tissue directly from the tumor mass. Without both normal and tumor tissue from the same specimen, the interpretation of mass spectra from margin samples can be challenging. Method development for assessing margins without measurement of the corresponding tumor samples is currently ongoing. Moreover, it is important to note that the dimensions of each of the surgical margin specimens that we sampled from the lumpectomy specimen represent only a small portion of the total surface present at the specimen margins, significantly reducing the likelihood of detecting positive margins should tumor be present. Despite this potential limitation, the endpoint analysis for our studies is to evaluate the sensitivity, specificity, and robustness of rapid ambient mass spectrometry in providing molecular information as a complement for expert histopathological tissue evaluation. We continue to accrue patients and will complete an analysis of surgical margins for 42 patients at the conclusion of the study. Therefore, although intraoperative MS is a goal for many in the field, the selection of a patient population designed to minimize potential confounding factors such as neoadjuvant therapy in initial trials, and clinical needs for histopathological diagnosis can limit specimen allocation for such studies.

Prior to ambient MS analysis, freshly resected BCS specimens needed to be allocated and processed, albeit in a minimal fashion. The two primary advantages of using fresh tissue, rather than frozen tissue or ex vivo mastectomy specimens, is the speed with which the analysis can be conducted and the ability to limit metabolic changes that can result from the freeze-thaw of samples or post-surgical time-dependent changes. Despite improvements in speed and reproducibility of the analysis itself, clinical logistics complicated our ability to perform real-time analysis in the operating room. Specifically, to localize biopsy markers and confirm tumor size, ex vivo mammography of the resected tumor specimen was needed, resulting in pre-analytical delay. Although specimens were maintained in a sterile container and imaged as quickly as possible, there was still a delay between surgical excision and MS analysis, which may have impacted cellular metabolite levels. Since we primarily measured fatty acids and glycerophospholipids, which are generally more stable than other metabolic intermediates such as glycolytic or anaplerotic substrates, organic acids, or acyl-CoA molecules, the impact of the pre-analytical interval may not be substantial. Nonetheless, reducing or at least better controlling the pre-analytical interval in addition to a systematic evaluation of analyte stability under the range of conditions of analysis is being considered for future studies, as well as for the remainder of this study.

In addition to pre-analytical metabolic artifact, another challenge in our study is sampling bias due to the heterogeneous nature of the breast resection tissue itself. While larger tissue specimens are generally preferred for most analytical methods, in this case, since only a small piece of the sample is analyzed, care must be taken to find a representative section of the tissue. To accomplish this, we avoided analyzing inked, bloody, or cauterized tissue to avoid sampling artifacts and prevent ion suppression, though we did freeze the remainder of the specimen for future analyses. For those analyses, the distance between the tumor and each of the corresponding margins will be considered.

Finally, we encountered some issues with the sample preparation. In our previous intraoperative studies with human brain specimens, we found that the physical nature of the brain tissue itself allowed facile tissue smearing, not dissimilar to the squash preparation techniques used for the intraoperative cytologic analysis of brain tissue^32,33^. Also, in our previous DESI-MS frozen breast sample study, we had selected cases that demonstrated more fibro-fatty tissue, which was easier to work with since the higher water to fat content allowed for easier sectioning and analysis^29^. In this study, which required the use of fresh breast tissue, we found that samples were considerably more fatty than the fibro-fatty frozen breast samples we had previously used. This difference led to more difficult processing than we had experience with brain specimens. In terms of slide preparation, additional manual compression of the sample was needed to flatten the breast tissue compared to brain tissue.

One of the major advantages of using an ambient MS method such as LMJ-SSP MS was rapid analysis time, which is significantly shorter than most currently available pathological techniques and other clinical MS platforms. The most commonly used MS platform in the clinical laboratory is liquid chromatography-tandem mass spectrometry (LC-MS/MS). Though more mature and robust, LC-MS/MS often requires more involved sample preparation or chromatographic separation, which is generally prohibitive for real-time or near real-time surgical decisions. Even MALDI-TOF analysis, which has revolutionized the clinical microbiology laboratory workflow for microbial identification, still requires establishing of vacuum in the source, which can slow down analysis. Using LMJ-SSP MS, we were able to start collecting mass spectral data within seconds and robust full MS data, including fatty acids and lipids in minutes after receiving the sample. Additional advantages of performing this testing ex vivo include the ability to use solvents that are less biocompatible but more efficient for reproducible extraction, as well as the potential for using a single system to simultaneously serve a number of operating rooms, much as a frozen section room might, with faster turnaround time.

Although similar analytical speed can, in principle, be achieved using DESI-MS, we encountered difficulties when we attempted to use DESI-MS for characterizing the initial fresh breast tissue samples from this study. Notably, pressure from the angled spray caused the fresh tissue to shift along the surface of the slide, thus preventing the reproducible acquisition of spectra, a phenomenon we had not previously seen when using DESI-MS on fresh brain tissue samples. These shifts were likely due to the high adipose content of breast tissue. After testing several different surfaces to prevent the lateral movement of the tissue, we compared DESI-MS to LMJ-SSP MS and found the signal to be more stable using LMJ-SSP MS. Qualitatively we observed that LMJ-SSP MS provided a more consistent signal by using a perpendicular source.

A critical concern using the LMJ-SSP platform is the potential for sample carryover. Unlike some other ambient ionization methods such as liquid extraction surface analysis (LESA), the LMJ-SSP uses the same capillary for all samples. Further, if the microjunction is too close to the tissue, there is a possibility of contamination of the needle and subsequent carryover. To overcome this, we found that flushing the capillary thoroughly between samples using a glass slide with a small depression-containing solvent decreased the occurrence of carryover. Without this step, the possibility of carryover is high and could potentially have a negative impact on analysis. Further improvements to decrease carryover, as well as a formal carryover study, will be needed to optimize this platform for clinical care. Despite the limitations, LMJ-SSP provided a more consistent signal than DESI-MS and demonstrated potential as a tool for surgical guidance in this and/or an engineered form that would optimize the interface between the patient and the mass spectrometer.

Unlike clinical LC-MS/MS, which typically involves the use of a triple quadrupole mass spectrometer, we acquired data using an ion trap instrument. While triple quadrupole instruments are ideal for measuring specific, known analytes in complex biological matrices, ion trap mass spectrometers are preferred for profile analysis, as they allow for the collection of a discrete quantity of ions, in our case 70,000 ions every 50 ms, thus providing a more robust chemical signature of the tissue. By segmentally targeting first the 600 *m/z* range for two minutes, followed by the 200 *m/z* range for two minutes, we acquired a more robust profile of both phospholipids and fatty acids, respectively. One of the shortcomings of using an ion trap with the unit resolution, however, is that lower mass resolution can lead to lower molecular specificity and a concomitant decrease in sensitivity. For more robust metabolomics studies, a high mass resolution instrument such as FT-ICR MS, Orbitrap, or Q-TOF could help differentiate molecules with similar *m/z* values. However, these instruments can be considerably more expensive and less “rugged,” making them less practical for routine implementation in an operating room. Furthermore, as with all ion traps, the strength of the instrument is in generating a profile, but if quantification is needed for specific metabolites, there can be decreased precision and accuracy in complex biological matrices, as compared to triple quadrupole instruments. For this reason, incorporation of an internal standard or a panel of internal standards either on the tissue or in the solvent itself may provide more consistent results; such approaches that introduce standards are currently under development.

Unlike IDH-mutant gliomas where we can monitor the oncometabolite 2-HG, there is no universal oncometabolite that has been reproducibly identified across breast cancers. In fact, there is growing evidence that different breast cancers exhibit significantly different metabolic profiles^34,35^. Using SAM analysis in this preliminary work, we found five features that demonstrated an FDR < 0.001, and notably, all were in the lipid mass range. This is, perhaps, not surprising as the higher cellular content of the tumor would correlate with a higher content of membrane glycerophospholipids and sphingolipids. Five phospholipid species were found to be statistically significant as tumor-related peaks, including phosphatidylethanolamine (PE), phosphatidylserine (PS), and phosphatidylinositol (PI). Due to the potential ion suppression of the lipid precursor ion, three of the significant peaks were represented as the M + 1 isotopologue. To confirm the identification of these lipids, a polar tissue extraction was performed and followed by a tandem-MS analysis in the laboratory. The lipids observed included [PS (P-16:0/18:2)-H]^−^ (*m/z* 742.6), [PE (18:0/20:4)-H]^−^ (*m/z* 766.6), [PE (18:2/20:1)-H]^−^ (*m/z* 768.6), [PS (O-18:0/20:3)-H]^−^ (*m/z* 798.6), and [PI (18:1/20:4)-H]^−^ (*m/z 883*.6). The t-SNE plot presented in Fig. 5 shows a general tendency of separating the normal and tumor groups, nevertheless, three normal cases were projected closely to data points from the tumor group (Fig. 5), which as noted earlier could be a result of tumor cells in the regions designated as normal by gross visual inspection and palpation or indicative of field cancerization. Another interesting finding was the clustering of the two triple-negative cases, which would be consistent with the mass spectrometer finding differences between cancer subtypes^29^. More cases are needed to investigate this intriguing possibility.

There were notable differences between the signatures we observed in our prior study^29^ and the current one. Previous work published by our lab using DESI-MS revealed chemical signatures in breast tumors, most comprised of ions with *m/z* 281.2 and 394.1^29^. Interestingly, for most of our intraoperative cases based on fresh tissue analysis, we did not note an abundance of either of these peaks in most of the tumor samples. On the contrary, we saw higher levels of 281.2 in the normal tissue samples, particularly in regions of high adiposity. However, caution must be taken when comparing spectra acquired using different platforms and from samples processed in different ways. The nature of analyte desorption using DESI compared to extraction using LMJ-SSP is fundamentally different and, therefore, could preferentially extract different analytes. Secondly, the previous study analyzed frozen and sectioned samples, rather than fresh samples processed in smear preparations. Therefore, it is possible that the chemical changes that occur during the freeze-thaw process may have also influenced the profile. In fact, we have recently shown in a cell culture system that simply depriving the cells of media and exposing them to air for five minutes can significantly increase levels of 281.2^36^. Finally, the samples analyzed for the DESI-MS study were acquired by a breast pathologist from mastectomies and carefully selected to be more fibro-fatty. The samples we receive in the OR have a notably higher adipose content and partially transition from a solid to liquid phase when deposited on glass slides. Taken together, differences in tissue composition, extraction chemistry, as well as processing stability artifact prevent direct comparison of the two studies. Identifying and overcoming these differences have presented challenges that have required considerable optimization of our intraoperative workflow for the trial.

We performed the presented interim analysis to assess the effect of having to adjust the desorption/ionization technology during the first phase of a registered clinical trial, i.e., from DESI to LMJ-SSP, and to share lessons learned as others are embarking on similar studies. The analysis of tumor and normal regions of the lumpectomy specimens, despite resulting in a limited number of specimens, provided optimal controls to address the technology modification. Given the limited dataset in this preliminary evaluation, we opted for unsupervised analysis of the data to avoid overfitting a classification model. More specifically, we first applied the statistical technique SAM to the limited dataset which reduced the ~1000 peaks to 5 with a false-discovery rate (FDR) <0.001. The visualization of these results with t-SNE suggests that the 5 statistically significant features allow the separation of tumor from normal tissue, but additional data will be required to consider any findings as potential markers for tumor delineation. Despite the need to modify the interface to the mass spectrometer in the course of our clinical trial, the resulting data supports the effort to continue accrual and analysis. Ongoing areas of focus include further characterization of clinical trial samples and the detailed identification and characterization of several of the specific molecules we have recently identified using higher resolution MS techniques, as well as the incorporation of robust statistical approaches to classify mass spectra and visualize classification results in real-time in the operating room. Efforts are planned to determine the effect of sample handling and processing on lipid and small molecule stability. This study highlights both the advantages and logistical challenges of using continuous flow LMJ-SSP MS for sample processing and analysis. Moreover, although it would be ideal to find broadly applicable biomarkers for breast cancer, there is increasing evidence that each patient may have variable metabolomic profiles, as has been predicted by many, and is part of the impetus for personalized medicine. As such, additional approaches need to be developed to not only analyze but characterize in real-time and create “metabolic fingerprints” which can then be assessed in surgical margins.

## Methods

### Materials

Mass spectrometry grade acetonitrile (ACN), dimethylformamide (DMF), and water were obtained from Fisher Scientific (Pittsburgh, PA). Standard glass slides were used for analysis.

### Sample collection and processing

BCS specimens were collected from 21 subjects according to our approved IRB protocol (Dana-Farber Cancer Institute IRB) and all subjects in the study were consented prior to the procedure, as part of a Phase II clinical trial: *ClinicalTrials.gov Identifier NCT02335671- Evaluating Mass Spectrometry And Intraoperative MRI In The Advanced Multimodality Image Guided Operating Suite (AMIGO) In Breast-Conserving Therapy* (date of registration: January 12, 2015). Written informed consent was obtained from all subjects. Surgical margin specimens were allocated peri-operatively directly from the specimen from anterior, posterior, superior, inferior, lateral, and medial margins. To obtain tumor specimens, the lumpectomy specimen was first re-imaged by specimen radiography and if tumor diameter was greater than 1.5 cm, a representative specimen from the tumor core was allocated and analyzed in AMIGO. For comparison, normal representative tissue samples were also obtained during the grossing of the lumpectomy specimen in the frozen section room. For MS analysis, a small piece (approximately 2 × 2 × 2 mm) of representative tissue was placed directly onto a standard glass slide and manually smeared between two slides prior to analysis.

### Ambient MS analysis

LMJ-SSP MS was performed using a customized FlowProbe (Prosolia Inc., IN) on the smeared breast tissue samples directly on the glass slide. The slide was placed on a stage that was controlled by the OmniSpray 2D software (Prosolia Inc., IN). A 1:1 ACN:DMF solution was used for analyte extraction and ionization, with solvent delivered by syringe at 5 μL/min. The ionization spray was directed into an ion trap mass spectrometer (amaZon speed, Bruker Daltonics), set for negative ion mode detection according to previously published protocol. Mass spectra were acquired for an interval of two to four minutes with half of the time at a target ion mass of *m/z* 600, and a half at a target ion mass of *m/z* 200, to capture a more robust spectrum of both fatty acids and phospholipids. Spectra were acquired using trapControl software and analyzed using Data Analysis software (version 4.2, Bruker Daltonics). Averaged mass spectra of the full interval were used for further statistical analysis.

### Stimulated Raman scattering (SRS) and second harmonic generation (SHG) microscopy

For two of the subjects, corresponding specimens were also analyzed by Stimulated Raman scattering (SRS) as follows. The principle and setup of the label-free stimulated Raman scattering (SRS) microscope have been previously described^37^. The laser source for the SRS microscope was a dual-color narrow-band one-box laser (picoEmerald, APE), providing the tunable pump beam (720–990 nm, pulse width 5–6 ps) and the Stokes beam at a fixed wavelength (1064 nm, pulse width, seven ps). Imaging was realized through raster-scanning of the tightly focused laser beam across the sample using a water-immersion objective (XL PLAN N ×25, NA 1.05; Olympus) on a modified confocal microscope platform (FV300, Olympus Inc.). The Raman shifts for SRS imaging were defined by the frequency difference between the pump and Stoke beams. By tuning the pump beam central wavelengths, multi-color imaging could be realized. The laser source was remotely controlled through an RS-232 interface. A single field of view (FOV) of laser-scanning imaging was 350 × 350 μm. Each FOV was acquired with 1024 × 2014 pixels in about ~4 s. Mosaic imaging was conducted using an automated stage (MS2000, ASI) with partial overlapping (~10%). To realize high-sensitive SRS imaging free from laser noise, the Stokes beam was amplitude-modulated at 10 MHz using an electro-optic modulator (EOM, Thorlabs), and the modulation transferred to the pump beam (i.e., stimulated Raman loss) was detected using a home-built all-analog lock-in amplifier. A Si photodiode detector was used to detect the pump light intensity. Labview programming (National Instruments Inc.) integrated the whole system for automatic multi-color and large-tissue image acquisition. Lipids were imaged (green) at 2854 cm^−1^ attributed to CH_2_ stretching vibration from the fatty acid long chains and proteins were imaged (blue) at 2940 cm^−1^ mainly attributed to CH_3_ vibration of the chemical bonds in the amino acids. Imaging resolution is ~400 nm. Scale bar, 350 μm.

Second-harmonic generation (SHG) imaging of collagen in the tissue samples was performed on the same microscopic platform as SRS. The OPO beam was tuned at 800 nm for SHG excitation and the 1064-nm laser beam was blocked. A dichroic mirror at 45 degrees was placed before the objective to pick the backward SHG signals from the sample. A short pass filter (FES0750, Thorlabs Inc.) and a bandpass filter (FB400-10, Thorlabs Inc.) was used to block both the excitation beam and two-photon fluorescence emissions in front of a photomultiplier tube (PMT) for SHG signal detection. Note that multi-color SRS images and SHG images were collected sequentially from the same tissue samples.

### Feature extraction and dimensionality reduction

Spectra were first aligned using the MATLAB function *mspalign*, in which a reference vector of common *m/z* values was determined, to which each individual spectrum was subsequently aligned. Following alignment, each individual spectrum was normalized to total ion count (TIC). An advanced statistical method termed significant analysis of microarray (SAM)^38^ was used to identify discriminating *m/z* features that could distinguish between normal and tumor spectra. We have used the publicly available *samr* package^39^ in which a two-class unpaired test with 6000 permutations of repeated measurements was used to identify a cut-off threshold (delta) that would achieve the lowest false discovery rate (FDR). The SAM features that achieved a false-discovery rate (FDR) <0.001 were extracted and considered significant. The non-linear dimensionality reduction method of t-SNE^40^ was applied to those extracted features to project the spectra into a 2D space. The t-SNE map was constructed in an unsupervised manner, without passing any prior information about the class labels. In this reduced feature space, spectra with similar feature profiles were projected close to each other while dissimilar ones were projected far apart. This allows visualization of the discriminating capabilities of those extracted features and facilitates data clustering^41,42^. Data points in the t-SNE map were then labeled based on their class category (normal or tumor), enabling visual assessment of their separability. The t-SNE map was also colored based on the ion expression of each of the *m/z* SAM features. Spectral distribution and hierarchical clustering using Euclidean distance and Ward clustering algorithm among samples was generated using Metaboanalyst^43^ and represented as a heat map.

### Reporting summary

Further information on research design is available in the Nature Research Reporting Summary linked to this article.

## Supplementary information

Supplementary Information

Supplementary Data 1

Reporting Summary

## Data Availability

The data generated and analyzed during this study are described in the following data record: 10.6084/m9.figshare.14959383^44^. The de-identified lipidomics data are contained in the Excel spreadsheet ‘SI_Table_Breast_MassSpec_Aligned_TIC.xlsx’, which is shared publicly as part of the figshare data record. A copy of the same data is also available as Supplementary Table 1 of this article.
