# Supplementary material for: Interim clinical trial analysis of intraoperative mass spectrometry for breast cancer surgery
Source: NPJ Breast Cancer. 2021 Sep 9;7:116. doi: 10.1038/s41523-021-00318-5 (PMC8429658; doi:10.1038/s41523-021-00318-5)
Supplement: Supplementary file 1 — Supplementary Information [file 41523_2021_318_MOESM1_ESM.pdf]

Supplementary Data 1. Aligned and total ion current (TIC)-normalized LMJ-SSP MS spectra of breast tissue samples.
