# Supplementary material for: Interim clinical trial analysis of intraoperative mass spectrometry for breast cancer surgery
Source: NPJ Breast Cancer. 2021 Sep 9;7:116. doi: 10.1038/s41523-021-00318-5 (PMC8429658; doi:10.1038/s41523-021-00318-5)
Supplement: Supplementary file 3 — Reporting Summary [file 41523_2021_318_MOESM3_ESM.pdf]

nature research

Corresponding author(s): Nathalie Agar
NPJBCANCER-00658R3

Reporting Summary

Nature Research wishes to improve the reproducibility of the work that we publish. This form provides structure for consistency and transparency in reporting. For further information on Nature Research policies, see Authors & Referees and the Editorial Policy Checklist.

Statistical parameters

When statistical analyses are reported, confirm that the following items are present in the relevant location (e.g. figure legend, table legend, main text, or Methods section).

n/a	Confirmed	
☐	☒	The exact sample size (*n*) for each experimental group/condition, given as a discrete number and unit of measurement
☐	☒	An indication of whether measurements were taken from distinct samples or whether the same sample was measured repeatedly
☐	☒	The statistical test(s) used AND whether they are one- or two-sided<br>*Only common tests should be described solely by name; describe more complex techniques in the Methods section.*
☐	☒	A description of all covariates tested
☐	☒	A description of any assumptions or corrections, such as tests of normality and adjustment for multiple comparisons
☐	☒	A full description of the statistics including central tendency (e.g. means) or other basic estimates (e.g. regression coefficient) AND variation (e.g. standard deviation) or associated estimates of uncertainty (e.g. confidence intervals)
☐	☒	For null hypothesis testing, the test statistic (e.g. *F*, *t*, *r*) with confidence intervals, effect sizes, degrees of freedom and *P* value noted<br>*Give P values as exact values whenever suitable.*
☒	☐	For Bayesian analysis, information on the choice of priors and Markov chain Monte Carlo settings
☐	☒	For hierarchical and complex designs, identification of the appropriate level for tests and full reporting of outcomes
☒	☐	Estimates of effect sizes (e.g. Cohen's *d*, Pearson's *r*), indicating how they were calculated
☐	☒	Clearly defined error bars<br>*State explicitly what error bars represent (e.g. SD, SE, CI)*

Our web collection on statistics for biologists may be useful.

Software and code

Policy information about availability of computer code

Data collection	The slide was placed on a stage which was controlled by the customized OmniSpray 2D software (Prosolia Inc., IN). The ion trap mass spectrometer (amaZon speed, Bruker Daltonics) was controlled and mass spectral data were collected using the trapControl software.
Data analysis	Data analysis was performed using Data Analysis software (version 4.2, Bruker Daltonics), MATLAB (version 2018a, Natick, MA, USA), and R Studio (version 3.5.0) a free software environment for statistical computing and graphics

For manuscripts utilizing custom algorithms or software that are central to the research but not yet described in published literature, software must be made available to editors/reviewers upon request. We strongly encourage code deposition in a community repository (e.g. GitHub). See the Nature Research guidelines for submitting code & software for further information.

Data

Policy information about availability of data

All manuscripts must include a data availability statement. This statement should provide the following information, where applicable:
- Accession codes, unique identifiers, or web links for publicly available datasets
- A list of figures that have associated raw data
- A description of any restrictions on data availability

The data generated and analyzed during this study are described in the following data record: https://doi.org/10.6084/m9.figshare.14959383. The de-identified

lipidomics data are contained in the Excel spreadsheet 'SI_Table_Breast_MassSpec_Aligned_TIC.xlsx', which is shared publicly as part of the figshare data record. A copy of the same data is also available as Supplementary Table 1 of this article.

Field-specific reporting

Please select the best fit for your research. If you are not sure, read the appropriate sections before making your selection.

☒ Life sciences ☐ Behavioural & social sciences ☐ Ecological, evolutionary & environmental sciences

For a reference copy of the document with all sections, see nature.com/authors/policies/ReportingSummary-flat.pdf

Life sciences study design

All studies must disclose on these points even when the disclosure is negative.

Sample size	BCS specimens were collected from 21 subjects according to our approved IRB protocol (Dana-Farber Cancer Institute IRB) and all subjects in the study were consented prior to the procedure, as part of a Phase II clinical trial: ClinicalTrials.gov Identifier NCT02335671- Evaluating Mass Spectrometry And Intraoperative MRI In The Advanced Multimodality Image Guided Operating Suite (AMIGO) In Breast-Conserving Therapy (date of registration: January 12, 2015).
Data exclusions	No data were excluded
Replication	Two glass slides are used to smear the tissue, one is analyzed in the operating room and the second one is preserved and analyzed in the laboratory to compare results. While we could not reliable analyze with DESI which was the original approach, we could replicate results with surface liquid extraction.
Randomization	There was no randomization for this study. One of the endpoint is to evaluate mass spectrometry in delineating tumor margins during surgery. For this midpoint analysis, the tumor and normal specimens were analyzed. All margins will be analyzed at completion of the trial.
Blinding	Blinding was not possible as the scientist is working in the operating room with the surgeon to analyze specific specimens

Reporting for specific materials, systems and methods

Materials & experimental systems

n/a	Involved in the study	
☐	☒	Unique biological materials
☒	☐	Antibodies
☒	☐	Eukaryotic cell lines
☒	☐	Palaeontology
☒	☐	Animals and other organisms
☐	☒	Human research participants

Methods

n/a	Involved in the study	
☒	☐	ChIP-seq
☒	☐	Flow cytometry
☒	☐	MRI-based neuroimaging

Unique biological materials

Policy information about availability of materials

Obtaining unique materials	The Prosolia source and the amaZon speed ion trap mass spectrometer are standard commercial sources. The clinical samples are considered unique materials that were only available under the a Phase II clinical trial: ClinicalTrials.gov Identifier NCT02335671- Evaluating Mass Spectrometry And Intraoperative MRI In The Advanced Multimodality Image Guided Operating Suite (AMIGO) In Breast-Conserving Therapy (date of registration: January 12, 2015).

Human research participants

Policy information about studies involving human research participants

Population characteristics	All 21 patients were treatment naive females over 18 years of age, with invasive breast cancer who were breast conservation therapy candidates at presentation.
Recruitment	Participants were recruited at a clinic visit by their treating surgeon co-investigator. Eligible patients were introduced to the study by their care team and, if interested, met with a member of the research team for further discussion and screening. If

agreeable to participate, the patient and surgeon went through the informed consent form together and sign were applicable.
